# Isolation and Characterization of Cross-Amplification Microsatellite Panels for Species of *Procapra* (Bovidae; Antilopinae)

**DOI:** 10.3390/ijms13078805

**Published:** 2012-07-16

**Authors:** Jing Chen, Chunlin Li, Ji Yang, Zhenhua Luo, Songhua Tang, Feng Li, Chunwang Li, Bingwan Liu, Zhigang Jiang

**Affiliations:** 1 Key Laboratory of Animal Ecology and Conservation Biology, Institute of Zoology, Chinese Academy of Sciences, No. 1 Beichen West Road, Beijing 100101, China; E-Mails: jingchen@ioz.ac.cn (J.C.); liclecology@gmail.com (C.L.); yangji@ioz.ac.cn (J.Y.); luozh@ioz.ac.cn (Z.L.); tangsh@ioz.ac.cn (S.T.); fengli@ioz.ac.cn (F.L.); licw@ioz.ac.cn (C.L.); 2 Graduate School of the Chinese Academy of Sciences, No. 19 (A) Yuquan Road, Beijing 100049, China; 3 School of Resources and Environmental Engineering, Anhui University, Hefei 230000, China; 4 College of Wildlife Resources, Northeast Forestry University, Harbin 150040, China; E-Mail: liubw1@163.com

**Keywords:** *Procapra*, genetic markers, microsatellite, cross-amplification, isolation strategy

## Abstract

The three *Procapra* species, Tibetan gazelle (*P. picticaudata*), Mongolian gazelle (*P. gutturosa*) and Przewalski’s gazelle (*P. przewalskii*) are endemic to Asia. Several intraspecific genetic issues have been studied with species-specific microsatellite loci in these Asian gazelles. However, cross-species microsatellite panels are absent, which inhibits comparative conservation and evolutionary studies of the *Procapra*. In this study, we isolated 20 cross-species microsatellite loci for *Procapra* from both related species and the genomic library of *P. przewalskii*. Fifty-three samples of the three gazelles were used to characterize the markers. Allele numbers ranged from three to 20, with a mean of 7.93 per locus. Observed heterozygosity (*H*_O_) averaged 0.680 and expected heterozygosity (*H*_E_) 0.767. The mean polymorphic information content (PIC) was 0.757 for *P. picticaudata*, 0.803 for *P. gutturosa* and 0.590 for *P. przewalskii*. Nine loci were significantly deviated from Hardy-Weinberg (H-W) equilibrium in the three species. Significant linkage disequilibrium was detected in four pairs of loci in *P. przewalskii*, five pairs in *P. gutturosa* and 51 pairs in *P. picticaudata*. Considering the abundance of published loci and their high success rates of cross-amplification, testing and utilization of loci from related species is efficient for wild species of Bovidae. The cross-species microsatellite loci we developed will facilitate further interspecies genetic studies in *Procapra*.

## 1. Introduction

Microsatellites (SSRs) are simple tandem repeated DNA sequence elements of one to six bases distributed in all prokaryotic and eukaryotic genomes [1,2]. Microsatellites are powerful and versatile genetic markers for their high degree of variability induced by replication slippage [3–6]. They can be used for studies of estimating genetic diversity [7,8], detecting gene flow [9], measuring genetic differentiation [10], rebuilding genetic structure [11], tracking the biological history of populations [12], distinguishing individuals [13,14], and analyzing pedigree relationship [15]. However, one drawback restricts the utilization of microsatellites: Primer sets must be developed or tested freshly for those species, which are studied for the first time [16]. A possible reason for this is that primer annealing might fail for the high evolutionary rates of flanking regions [16–18].

**Figure 1:**
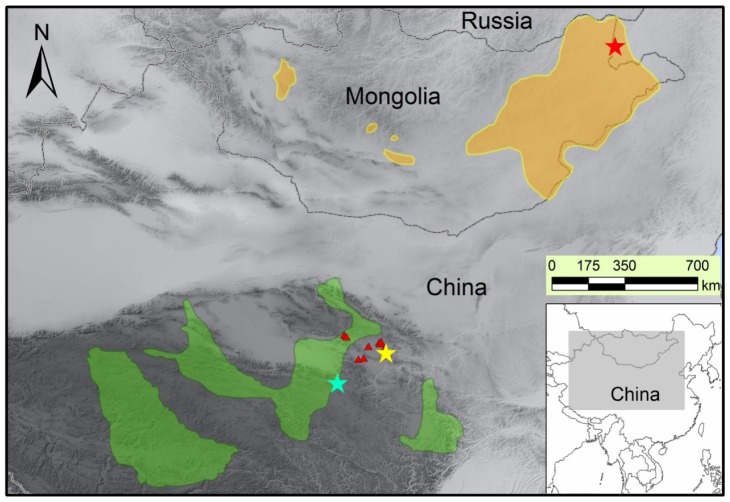
Map of the distribution ranges and sampling locations of *Procapra* (*P. picticaudata*, *P. gutturosa* and *P. przewalskii*).

*Procapra* are ancient Asian antelopes that are important in terms of their phylogenetic and conservation position. The group split from the tribe Antilopini at the first radiation event of gazelles during 11–12 Ma [19], but their populations are threatened by extinction making them a focus of global conservation [20,21]. The genus has three existent species: Tibetan gazelle (*P. picticaudata*) that spreads over the Qinghai-Tibet Plateau in China with a small population in Ladakh and Sikkim [22–24], the Mongolian gazelle (*P. gutturosa*) that has survived in Mongolia and inner-Mongolia of China [25,26], and the Przewalski’s gazelle (*P. przewalskii*) that could be found in the North and West of China 150 years ago, whose range, however, has recently shrunk to fragmented patches around Qinghai Lake [27–29]. Recently one population of *P. przewalskii* has been found living sympatrically with *P. picticaudata* in upper the Buha River valley [30,31]. According to locations of type specimen, *P. przewalskii* used to live sympatrically with *P. gutturosa* in inner Mongolia 60 years ago as well [32] (Figure 1). Populations of the three species are declining and are threatened by hunting, excessive livestock grazing, and habitat loss or fragmentation [26,33–36]. They are listed as Category I (*P. przewalskii*) or Category II (*P. picticaudata*, *P. gutturosa*) of the National Key Protected Wild Animals in China, and as “Near Threatened” (*P. picticaudata*), “Least Concern” (*P. gutturosa*) or “Endangered” (*P. przewalskii*) in the IUCN Red List [20,37]. Their threatened status is drawing attention of both scientific and conservation communities, and field conservation projects have been launched for the endangered Przewalski’s gazelle [27].

The green area represents the distribution range of *P. picticaudata* and the orange area represents the distribution of *P. gutturosa*. The red triangles represent the scattered distributed patches of *P. przewalskii*. The blue, red and yellow pentagrams indicate sampling locations of *P. picticaudata*, *P. gutturosa* and *P. przewalskii,* respectively. The shaded rectangle on the inset indicates the location of the main map in China.

Population genetics studies on these Asian gazelles have been performed and they provide recommendations for conservation and management of *Procapra*. Zhang *et al*. demonstrated that *P. picticaudata* had divided into three geographic populations according to the results of phylogenetic analysis of mtDNA [38]. Yang *et al*. employed 13 microsatellite loci and revealed that populations of *P. przewalskii* possessed a moderate nuclear genetic diversity [39], and that anthropogenic landscape was one of the main factors that shaped their genetic structure [34]. A study of *P. gutturosa* showed high mitochondrial genetic diversity with no obvious genetic structure [40]. All the previous genetic studies focused on single species and obtained intraspecific findings, however, important issues about the speciation in *Procapra* can only be addressed using interspecific comparisons. For example, how did the three species diverge or coexist in the contiguous or sympatric habitats? Is there any evidence of interspecific hybridization, and if not, what reproductive isolation mechanisms are there? The distribution range of *P. przewalskii* overlaps with that of *P. picticaudata*, but in fact it was split from *P. gutturosa* phylogenetically, how did this happen in evolution [41]? To find answers for these interspecific issues, it is essential to develop new microsatellite markers, which are suitable for cross amplification among the three species of *Procapra*.

Zhang developed nine microsatellite loci for *P. picticaudata* among which three were shared with *P. przewalskii* [42,43]. Yang isolated ten more microsatellite primers for *P. przewalskii* from related species [41]. However, we do not know whether these loci can be used across the three *Procapra* species. In this study, we tested their cross-amplification utility. In addition, we employed two methods, testing loci from related species and the construction of an enriched genomic library, to isolate more cross-species microsatellite primer sets for the three species of *Procapra*.

## 2. Results and Discussion

### 2.1. Isolation and Characterization of Cross-Amplification Microsatellite Loci

Five of 13 loci of *P. przewalskii* were amplified robustly and proved to be polymorphic in *P. picticaudata* and *P. gutturosa*. Eight of the 13 loci from four related species were amplified successfully in the three *Procapra* species and six of them were highly polymorphic. Regarding the method of construction of the genomic library, 300 clones were initially obtained, of which 110 positive clones were screened successfully by PCR, and finally 49 of them contained repeat motifs. Nineteen primer pairs were designed and nine of them yielded specific products among the three species. The nine loci were labeled and all of them were proved to be of high polymorphism. In total, 20 microsatellite loci suitable for the three species were isolated and characterized by the two methods (Table 1).

Allele numbers per polymorphic locus across the three species ranged from 3 to 20, with a mean of 7.93 (8.00 for *P. picticaudata*, 11.05 for *P. gutturosa* and 4.75 for *P. przewalskii*). The average observed heterozygosity (*H*_O_) was 0.680 (0.663 for *P. picticaudata*, 0.784 for *P. gutturosa*, and 0.593 for *P. przewalskii*). Expected heterozygosity (*H*_E_) averaged at 0.767 (0.804, 0.841 and 0.654 for *P. picticaudata*, *P. gutturosa* and *P. przewalskii*, respectively). The polymorphic information content (PIC) ranged from 0.271 to 0.907, and averaged 0.757 for *P. picticaudata*, 0.803 for *P. gutturosa* and 0.590 for *P. przewalskii* (Tables 2–4). After adjustment by the Bonferroni correction [48], nine loci were significantly deviated from Hardy-Weinberg (H-W) equilibrium, among which six were found in *P. picticaudata*, one in *P. gutturosa*, and four in *P. przewalskii*. Significant linkage disequilibrium was detected in four pairs of loci in *P. przewalskii* (AC29-AC230, AC1-AC77, AC77-CSSM43 and AC230-VH34), five pairs in *P. gutturosa* (HD28-MAF23, AC1-VH34, TANXAN-15-AF5, AC29-AC299 and BM4505-VH34) and 51 pairs of loci in *P. picticaudata*.

### 2.2. General Discussion of Results

#### 2.2.1. Isolation Strategies for Polymorphic Microsatellite

In our study, 39 percent of 13 primer pairs used in *P. przewalskii* were applicable in the other two species, and 46 percent of 13 primer pairs from four other species were usable in all the species of *Procapra*. Sequence conservation of the flanking regions of microsatellite loci allowed primer pairs designed for one species to be shared with closely related taxa [49,50]. Among the Bovidae species, cross-species amplification of microsatellite primer pairs of *Bos taurus* showed 30 percent success rate in *Capra hircus* [51] and 40 percent in *Ovis aries* [49]. Considering the absence of sequence screening and primer designing, developing microsatellite loci from related species is economical both in time and funds. However, for some target species without enough usable reference primers or sequences, construction of a genomic library is the only way to develop microsatellite loci [52,53]. Notwithstanding, cloning efficiency is always low in traditional isolation processes. Among the primer notes published in *Molecular Ecology* from 1999 to March 2001, which used traditional genomic library protocols, percentages of positive clones were as low as 0.04 percent, and averaged at 1.67 percent in mammals [16]. Thus, many optimized protocols and alternative approaches were proposed to solve the problem [16,52]. In our study, selective hybridization and enrichment were applied to increase cloning efficiency and finally 16 percent positive clones were obtained.

Recently, several advanced approaches of isolating microsatellites were developed. Despite the advantages, there are also some limitations. For example, methods of screening expressed sequence tags (ESTs) database [54–56] rely on published data, which are always unavailable for less studied species. Outputs of microsatellite loci through the newly developed next-generation sequencing technologies are of larger quantity but are usually redundant for studies of wildlife molecular ecology [57,58].

In summary, as there are almost 500 microsatellite markers of Bovidae deposited in the database of *Molecular Ecology Resource* till May 2012 [59], cross-amplification of microsatellite primers from related species seems to be feasible and economical for wild species of Bovidae.

#### 2.2.2. Genetic Diversity of the Three *Procapra* Species

In our study, both *P. picticaudata*, and *P. gutturosa* showed a high genetic diversity with high expected heterozygosity (*H*_E_ = 0.804 and 0.840), which was consistent with the results of Zhang [42] (*H*_E_ = 0.788 for *P. picticaudata*) and Sorokin *et al*. (5.85 ± 2.92 percent of average nucleotide diversity for *P. gutturosa*) [40], indicating high representative power of our primer sets. However, the number of alleles (*N* = 4.75), observed heterozygosity (*H*_O_ = 0.593) and expected heterozygosity (*H*_E_ = 0.654) of *P. przewalskii* were all significantly lower than for both *P. picticaudata*, and *P. gutturosa*, manifesting lower genetic diversity in the endangered gazelle. Yang *et al.* also got similar results (*H*_O_ = 0.525 and *H*_E_ = 0.552) in the study of genotyping and analyzing 169 individuals from nine subpopulations of *P. przewalskii* [39]. Possible reasons are that *P. przewalskii* has recently experienced a severe population decline and a genetic bottleneck [26,39,43]. Our result highlights the conservation emergency of the endangered *P. przewalskii* again.

## 3. Experimental Section

### 3.1. Sample Collection and Genomic DNA Extraction

Fifteen skin and muscle samples of *P. przewalskii* in one subpopulation (Hudong) were plucked from carcasses, which died from natural predation and disease. One blood sample of the Bird Island subpopulation was provided by the Qinghai Lake National Nature Reserve. Sixteen muscle samples of *P. picticaudata* were collected from local hunters in Dulan, Qinghai Province. Twelve muscle samples of *P. gutturosa* came from body remains killed by wolves in grasslands around Arihashate Manzhouli Customs, Inner Mongolia in 2004, and ten additional samples, which were confiscated as smuggled goods in 2010, were provided by A Day Hasha Te Manzhouli Customs (Figure 1).

Genomic DNA was extracted from the samples using the Universal Genomic DNA Extraction Kit Ver.3.0 (TaKaRa) on a Clean Bench. Extraction followed the manufacturer’s instructions except that digestion was prolonged by two hours to make sure that dried muscle or skin samples were fully digested. Genomic DNA yield was checked by gel electrophoresis with 1.5 percent of agarose.

### 3.2. Isolation of Microsatellite Markers

#### 3.2.1. Cross-Amplification of Microsatellite Loci from Related Species

Thirteen primer pairs developed for *P. przewalskii* by Yang [43] including the three loci which were already known to be usable in *P. picticaudata* [42] were tested in both *P. picticaudata* and *P. gutturosa*. Thirteen microsatellite primer pairs from four related species (*Gazella granti* [45], *Madoqua kirkii* [60], *Gazella dorcas* [61] and *Antilocapra americana* [44]) with long repeat motifs and high polymorphism were chosen and tested in all the three *Procapra* species by cross-species amplification.

All the PCR reactions were performed in a 10 μL volume containing 1× PCR buffer, 2.0 mM MgCl_2_, 0.2 mM of each dNTPs, 0.5 μM of each primer, 0.25 units Hotstart Taq DNA polymerase (TaKaRa) and 10 ng genomic DNA. Amplification cycles were carried out on a Thermo Hybaid MBS 0.2 S PCR Thermal Cycler (Thermo Fisher Scientific). The optimized touchdown PCR thermal cycling profile was: 10 min at 95 °C for initial polymerase activation, followed by 14 or 16 cycles of 30 s at 95 °C, 45 s at 64 °C and 1 min at 72 °C, with the annealing temperature decreasing 1 °C per cycle, then 35 cycles of 30 s at 95 °C, 45 s at 50 °C or 48 °C and 1 min at 72 °C, and a final extension step at 72 °C for 30 min. PCR products were visualized on two percent of agarose gel. Loci which produced robust and specific bands in all the three species were sequenced to make sure that the products contained microsatellites. Finally, the suitable loci were labeled with a fluorescent dye (6′-FAM, TAMARA, or HEX) on the 5′ end of forward primer.

#### 3.2.2. Construction of Enriched Genomic Library

To get further cross-species microsatellite loci for *Procapra*, an enriched genomic library of *P. przewalskii* was constructed according to Techen *et al.* [53], Zane *et al*. [16] and Liu *et al*. [62] with optimization of the processes. Briefly, genomic DNA extracted from the blood samples was digested by *Sau*3A I (TaKaRa), and the products were ligated to a phosphorylated adaptor (Oligo A 5′-GCGGTACCCGGGAAGCTTGG-3′, Oligo B 5′-pGATCCCAAGCTTCCCGGGTACCGC-3′) designed by Hamilton *et al*. [63]. Fragments ranging from 200 bp to 1000 bp were selected and hybridized with the biotin-labeled (AC)_15_ probe (Life Technologies). Fragments containing repeats were captured by streptavidin-coated magnetic beads (Promega). After elution and PCR enrichment, the target fragments were inserted into pMD18-T vectors (TaKaRa) and transformed to a *E. coli* JM109 strain (TransGen Biotech). Clones that contained AC repeat were screened by PCR reaction and sequenced with an ABI PRISM 3730XL DNA sequencer (Applied Biosystems). Primers were designed with Primer Premier 6.0 (Premier Biosoft International) for the appropriate sequences which contained large numbers of repeats and long enough flanking regions. The primers which produced single bands with the right size were labeled with fluorescent dye (6′-FAM, TAMARA, or HEX).

### 3.3. Polymorphisms Assessment

Polymorphism and the performance of all the chosen loci were assessed by 15 samples of *P. przewalskii* (excluding the blood sample from the Bird Island subpopulation), 16 of *P. picticaudata* and 22 of *P. gutturosa*. PCR reaction was performed in the previous touchdown profiles with different annealing temperatures for each primer pair. Products were resolved with an ABI PRISM 3730XL DNA sequencer (Applied Biosystems) and scored by GeneMarker V1.7 (SoftGenetics). Genepop ver.4 [64] was used to calculate the number of alleles, observed and expected heterozygosity (*H*_O_ and *H*_E_). The frequency of null alleles and the polymorphic information content (PIC) were estimated by CERVUS 2.0 [65]. Tests for deviation from H-W equilibrium and linkage disequilibrium were performed in Genepop ver.4 and corrected for multiple comparisons using a sequential Bonferroni correction.

## 4. Conclusions

This study is the first attempt to isolate cross-species microsatellite loci for *Procapra*. The 20 microsatellite primer pairs isolated by two methods are usable in both intraspecific and interspecific research of *Procapra* species. These microsatellites can be utilized in studies of genetic structure, genetic diversity, hybridization, speciation, and evolution of *Procapra*, making a contribution to conservation and management of the three Asian gazelles. Our study also suggests that obtaining primers from related species may be a good strategy for the development of microsatellite loci for bovid species.

## Figures and Tables

**Table 1:** Twenty microsatellite loci isolated for three species of *Procapra*.

Locus	Repeat motif	Primer sequences (5′–3′)	Size range (bp)	Tm (°C)	Source
AC1 #	(AC)_14_	F: TTGGCAGGTGGATTATTTAC R: TGGTTGTCAATGGAAGGAA	171–199	50	This study
AC29 #	(AC)_14_	F: AGGACGGCACTTAAACTTATG R: TATCATTGTCAGGCTTCTCT	169–198	50	This study
AC35 #	(AC)_12_GAAGTATA T(AC)_4_	F: TGGACAGAAGAGCGTAATG R: TCCTTGTGGCTGAGTAGTA	210–222	50	This study
AC77 #	(GT)_13_	F: CACAGTCTCTTCTCATAATGC R: CGGATTCTTTACCTCATACAC	147–161	50	This study
AC91 #	(AC)_14_	F: TTGGTCGTACTGACTGGTA R: GGAGTGACTGAGGACAGA	176–200	50	This study
AC170 #	(AC)_19_	F: TCTCAAGAGGCAGGTCAG R: GATTCCTTTGGCTCCTAGAAG	230–260	50	This study
AC230 #	(AC)_16_ATATGC (AC)_6_	F: TGGCTGAGCAACTAAGAG R: GGGAAATACTTGGGTAACAG	152–168	50	This study
AC244 #	(AC)_6_ (GT)_14_ (T)_5_G(T)_9_C(T)_9_	F: GGGATAGCAGAGAGTCAGA R: GGAAGGAACAATTAGGAGTATG	332–350	50	This study
AC299 #	(AC)_5_T(AC)_8_	F: CGGTGTTCATATAACAGATTCC R: GGTTGCTCAGTGGTCTCA	159–189	50	This study
Aam9 †	(GT)_15_	F: ATGTGGGAGACTTGATGATG R: AAGACTGGAGACTGGGATTATC	205–227	52	[44]
HDZ8 †	(AC)_14_	F: GACAAACACTCAGAAGGCAAAG R: GGTGGCAGGACTGAGCAAG	132–166	50	[45]
HDZ496 †	(AC)_15_	F: GTTTTTCCAGATGGTATTTTCCTC R: GTATTCGGCTGAAGGGACC	228–250	48	[45]
MAF23 †	(GT)_20_	F: GTGGAGGAATCTTGACTTGTGATAG R: GGCTATAGTCCATGGAGTCGCAG	124–160	50	[46]
VH34 †	(AC)_17_	F: TCGTAAGAGTGGACACAACTGAGCG R: CGCAGTATTTAGTCCTTTTAATAATGGC	81–101	50	[46]
BM4505 †	(ACAT)_4_(AC)_11_	F: TTATCTTGGCTTCTGGGTGC R: ATCTTCACTTGGGATGCAGG	240–258	48	[47]
AF5 ‡	(CA)_18_	F: GTGGGAAGAGATAGAGGAAGC R: GAGCCACAAGGCACAGCCAAC	135–157	51	[43]
BM1225 ‡	(CT)_13_TA(CA)_18_	F: TTTCTCAACAGAGGTGTCCAC R: ACCCCTATCACCATGCTCTG	231–275	50	[43]
CSSM43 ‡	(CA)_15_AT(CT)_5_	F: AAAACTCTGGGAACTTGAAAACTA R: GTTACAAATTTAAGAGACAGAGTT	246–268	48	[43]
RT1 ‡	(GT)_22_	F: TGCCTTCTTTCATCCAACAA R: CATCTTCCCATCCTCTTTAC	195–233	50	[43]
TEXAN-15 ‡	(CT)_9_TT(CT)_5_GCAG ATA(CA)_20_	F: TCGCAAACAGTCAGAGACCACTC R: TGGATGAGAAAGAAGAGCAGAGTTG	203–227	50	[43]

Tm, annealing temperature.

# Loci isolated by construction of genomic library.

† Loci from related species.

‡ Loci from *P. przewalskii*.

**Table 2:** Characterization of the twenty cross-species microsatellite loci in *P. picticaudata*.

Locus	No. of samples	No. of alleles	*H*_O_	*H*_E_	PIC	*P*_HW_	Null allele frequency
AC1	16	6	0.500	0.772	0.709	0.010	0.209
AC29	16	6	0.375	0.667	0.695	0.004	0.276
AC35	16	5	0.438	0.627	0.557	0.003	0.212
AC77	16	6	0.000	0.807	0.748	0.000 *	1.000
AC91	16	8	0.625	0.851	0.801	0.004	0.145
AC170	16	13	1.000	0.923	0.885	0.000 *	−0.057
AC230	16	7	0.688	0.815	0.757	0.013	0.064
AC244	16	6	0.625	0.593	0.546	0.836	−0.091
AC299	16	9	0.813	0.859	0.814	0.038	0.014
Aam9	16	7	0.813	0.829	0.776	0.252	−0.008
HDZ8	16	13	0.938	0.925	0.888	0.003	−0.026
HDZ496	16	11	0.875	0.905	0.865	0.077	0.005
MAF23	16	12	0.875	0.897	0.857	0.052	−0.003
VH34	16	8	0.625	0.857	0.810	0.002	* 0.145
BM4505	16	4	0.563	0.599	0.531	0.379	0.020
AF5	16	8	0.750	0.869	0.823	0.096	0.064
BM1225	16	11	0.688	0.919	0.881	0.000 *	0.123
CSSM43	16	3	0.938	0.643	0.552	0.029	−0.223
RT1	16	8	0.500	0.859	0.810	0.001 *	0.242
TEXAN-15	16	9	0.625	0.871	0.825	0.000 *	0.157

*H*_O_, observed heterozygosity; *H*_E_, expected heterozygosity; PIC, estimated polymorphic information content; *P*_HW_, probability of deviation for Hardy-Weinberg (H-W) proportions (*p*-value); Null allele frequency, estimated null allele frequency;

* loci which deviate from H-W equilibrium (after sequential Bonferroni correction, *p* = 0.0025).

**Table 3:** Characterization of the twenty cross-species microsatellite loci in *P. gutturosa*.

Locus	No. of samples	No. of alleles	*H*_O_	*H*_E_	PIC	*P*_HW_	Null allele frequency
AC1	22	12	0.864	0.904	0.872	0.010	0.009
AC29	22	14	0.909	0.927	0.898	0.573	−0.003
AC35	22	5	0.682	0.723	0.653	0.646	0.022
AC77	22	5	0.500	0.661	0.610	0.150	0.133
AC91	22	10	0.818	0.881	0.845	0.026	0.025
AC170	22	12	0.864	0.921	0.891	0.001 *	0.020
AC230	22	10	0.773	0.870	0.833	0.201	0.052
AC244	22	15	0.818	0.914	0.884	0.119	0.044
AC299	22	11	0.818	0.825	0.791	0.273	0.001
Aam9	22	12	0.682	0.883	0.850	0.037	0.120
HDZ8	22	12	0.818	0.793	0.759	0.610	−0.058
HDZ496	22	9	0.636	0.819	0.775	0.057	0.112
MAF23	22	14	0.955	0.928	0.900	0.018	−0.028
VH34	22	10	0.727	0.764	0.726	0.521	0.020
BM4505	22	4	0.500	0.602	0.542	0.075	0.063
AF5	22	14	0.955	0.886	0.856	0.814	−0.053
BM1225	22	20	0.818	0.935	0.907	0.008	0.056
CSSM43	22	8	0.864	0.804	0.754	0.981	−0.048
RT1	22	13	0.909	0.892	0.860	0.465	−0.024
TEXAN-15	22	11	0.773	0.884	0.850	0.091	0.063

*H_O_*, observed heterozygosity; *H_E_*, expected heterozygosity; PIC, estimated polymorphic information content; *P*_HW_, probability of deviation for H-W proportions (*p*-value); Null allele frequency, estimated null allele frequency;

* loci which deviate from H-W equilibrium (after sequential Bonferroni correction, *p* = 0.0025).

**Table 4:** Characterization of the twenty cross-species microsatellite loci for *P. gutturosa*.

Locus	No. of samples	No. of alleles	*H*_O_	*H*_E_	PIC	*P*_HW_	Null allele frequency
AC1	15	5	0.600	0.543	0.496	0.895	−0.079
AC29	15	5	0.933	0.786	0.721	0.470	−0.112
AC35	15	3	0.800	0.570	0.456	0.093	−0.200
AC77	15	5	0.333	0.816	0.755	0.000 *	0.400
AC91	15	4	0.667	0.559	0.491	1.000	−0.116
AC170	15	5	0.733	0.793	0.728	0.101	0.025
AC230	15	5	0.733	0.763	0.690	0.278	−0.002
AC244	15	4	0.333	0.715	0.635	0.000 *	0.368
AC299	15	5	0.467	0.749	0.686	0.005	0.231
Aam9	15	5	0.333	0.578	0.545	0.009	0.282
HDZ8	15	5	0.333	0.412	0.381	0.192	0.059
HDZ496	15	4	0.600	0.524	0.432	0.000 *	−0.103
MAF23	15	6	0.533	0.683	0.626	0.023	0.138
VH34	15	6	0.800	0.749	0.686	0.921	−0.041
BM4505	15	4	0.800	0.733	0.656	0.215	−0.071
AF5	15	3	0.267	0.301	0.271	0.009	0.105
BM1225	15	7	0.667	0.809	0.750	0.000 *	0.079
CSSM43	15	5	0.467	0.759	0.686	0.013	0.231
RT1	15	5	0.600	0.575	0.520	0.104	−0.026
TEAXAN-15	15	4	0.867	0.671	0.586	0.252	−0.150

*H_O_*, observed heterozygosity; *H_E_*, expected heterozygosity; PIC, estimated polymorphic information content; *P*_HW_, probability of deviation for H-W proportions (*p*-value); Null allele frequency, estimated null allele frequency;

* , loci which deviate from H-W equilibrium (after sequential Bonferroni correction, *p* = 0.0025).
